# Comparison between model global AWaRe-based quality indicators and quality indicators developed to assess the appropriateness of antibiotic prescribing in primary healthcare in South Africa

**DOI:** 10.3389/fpubh.2026.1847084

**Published:** 2026-07-03

**Authors:** Audrey K. Chigome, Aislinn Cook, Annie Heath, Filip Djukic, Yasmina Johnson, Sabiha Y. Essack, Adrian Brink, Marc Mendelson, Stephen M. Campbell, Brian Godman, Johanna C. Meyer

**Affiliations:** 1 Department of Public Health Pharmacy and Management, School of Pharmacy, Sefako Makgatho Health Sciences University, Pretoria, South Africa; 2 South African Vaccination and Immunization Center, Sefako Makgatho Health Sciences University, Pretoria, South Africa; 3 Antibiotic Policy Group, Institute for Infection and Immunity, City St. George's, University of London, London, United Kingdom; 4 Nuffield Department of Primary Care Health Sciences, University of Oxford, Oxford, United Kingdom; 5 Medicine Management, Laboratory and Blood Services Support, Department of Health and Wellness, Cape Town, South Africa; 6 School of Pharmacy, University of the Western Cape, Cape Town, South Africa; 7 Antimicrobial Research Unit, School of Health Sciences, University of KwaZulu-Natal, Durban, South Africa; 8 Division of Medical Microbiology, Faculty of Health Sciences, University of Cape Town, Cape Town, South Africa; 9 National Health Laboratory Service, Groote Schuur Hospital, Cape Town, South Africa; 10 Institute of Infectious Disease and Molecular Medicine, Faculty of Health Sciences, University of Cape Town, Cape Town, South Africa; 11 Division of Infectious Diseases and HIV Medicine, Department of Medicine, Groote Schuur Hospital, University of Cape Town, Cape Town, South Africa; 12 School of Health Sciences, University of Manchester, Manchester, United Kingdom

**Keywords:** antibiotic prescribing, antibiotic stewardship, antimicrobial resistance, primary healthcare, quality indicators, RAND/UCLA appropriateness method, South Africa, WHO AWaRe

## Abstract

**Background/objectives:**

Bacterial antimicrobial resistance represents a significant global public health challenge. Researchers from City St Georges, University of London, UK, developed global model sets of quality indicators, based on WHO AWaRe guidance, to improve future primary healthcare (PHC) prescribing. These were subsequently adapted to the PHC context in South Africa as an exemplar for developing countries. The study aimed to compare nationally adapted indicators in South Africa with global model indicators to improve future prescribing.

**Methods:**

Comparative analysis of the two sets of quality indicators, developed using the same Research and Development/University of California Los Angeles (RAND/UCLA) Appropriateness Method (RAM). For the 62 overlapping indicators rated in the second round of the RAM for both sets, the median appropriateness and feasibility ratings, and the level of consensus, were compared. The contextual appropriateness, feasibility, and adaptation of the indicators across public PHC facilities in South Africa were assessed to identify factors that could affect implementation.

**Results:**

Of the 62 overlapping indicators, only six (9.7%) were rephrased for the South African RAM to reflect local guidance. Fifty nine (95.2%) indicators were rated appropriate for the global RAM while all indicators (100%) were rated appropriate for South Africa. For the global RAM, 23 (37.1%) indicators were rated feasible while 60 (96.8%) indicators were rated feasible for South Africa. Seven (11.3%) indicators were rated both appropriate with agreement and feasible with agreement for the global RAM. Forty six (74.2%) indicators were rated both appropriate with agreement and feasible with agreement by the South African panelists, including seven indicators rated both appropriate and feasible by the global panel. The South African indicators adapted from the global set covered seven categories: respiratory tract infections, lower urinary tract infections, skin and soft tissue infections, diarrhea and enteric fever, bacterial eye infections, and dental infections, as well as general indicators.

**Conclusions:**

The South African indicators for public PHCs facilities, adapted from the model global set, show that local adaptation is imperative to reflect differences in national treatment guidelines between countries, prescribing cultures, epidemiology and infectious disease burden. Ongoing barriers include a reliance on paper-based records and poor-quality routine healthcare data.

## 1 Introduction

Bacterial antimicrobial resistance (AMR) represents a significant global public health challenge (1, 2), which disproportionately affects low-and middle-income countries (LMICs) (3–5). In the absence of effective interventions, AMR could result in 4.1 million deaths in Africa alone by 2050 (5). Inappropriate use of antibiotics is considered a key driver for resistance with 80%−90% of antibiotic use occuring in the primary care setting especially among LMICs (6–10). Optimizing use of antibiotics is essential to ensure patients are being treated with the right antibiotic, where needed.

Traditionally, antimicrobial stewardship (AMS) programmes in LMICs have mostly focused on hospital settings as there have been concerns with available trained personnel and resources to conduct these at the primary healthcare (PHC) level in low-resource settings (11–16). However, given primary care accounts for a large volume of antibiotic prescribing, and this setting is typically the first point of contact for patients with infectious diseases, there is an increasing focus on AMS in primary care (6, 17–22). In PHC settings, antibiotics are frequently prescribed empirically, often for self-limiting viral infections, due to diagnostic uncertainty, limited consultation time, and patient expectations along with a lack of access to updated clinical guidelines (19, 21, 23–25). Multiple studies conducted in LMICs consistently report high rates of inappropriate antibiotic prescribing in PHC settings, including unnecessary initiation of antibiotic therapy, incorrect antibiotic choices, high use of Watch antibiotics, sub-optimal dosing and excessive therapy duration (21, 25–31). There are similar concerns with high levels of inappropriate prescribing of antibiotics in primary care in South Africa, including for self-limiting viral infections (Supplementary Table S1) (27).

One of the World Health Organization's (WHO) eight priority AMR-related interventions for strengthening PHC-oriented health systems to combat AMR is ensuring that antimicrobials are used appropriately and according to updated evidence-based treatment guidelines (32). In view of this, a systematic assessment of antibiotic prescribing quality and appropriateness in PHC settings is critical, with audits and feedback surrounding agreed targets shown to improve future antibiotic prescribing (33–36). Quality indicators, defined as “*quantitative measures that provide information about the effectiveness, safety and/or people-centredness of care”*, have previously been used for evaluating the appropriateness of prescribing, including for antibiotics (37–40). It is recognized that appropriate and feasible evidence-based quality indicators are important for sustainable improvements in antibiotic stewardship in PHC settings. This is because they enable accurate measurement, and consequently improvement, of the appropriateness of current antibiotic use (33, 40, 41).

In South Africa, examples of indicators that have been used to assess the quality of antibiotic prescribing in PHC facilities are shown in Supplementary Table S2. This highlights the limited number of indicators that have been implemented to date at this level of care in South Africa.

The WHO developed the Access, Watch and Reserve (AWaRe) classification system, guiding the utilization of antibiotics, encouraging the preferential use of Access antibiotics and preserving those with high resistance potential, specifically those from the Watch and Reserve groups (42, 43). In 2022, the WHO developed the AWaRe antibiotic book to provide guidance on the use of the essential medicines list antibiotics for the treatment of the 35 common conditions in primary care and hospital settings (44, 45). To date, most quality indicators developed globally to improve future antibiotic use, do not incorporate treatment guidance provided in the WHO AWaRe antibiotic book (44–47). This is important, especially among LMICs, given concerns with the robustness of many national antibiotic guidelines in these countries (48).

As part of the Antibiotic Data to Inform Local Action (ADILA) project, researchers from City St Georges, University of London (SGUL), United Kingdom developed comprehensive global model sets of quality indicators based on the WHO AWaRe antibiotic book, which encompass primary and hospital care, as well as general indicators for antibiotic use (49, 50). Subsequently, these indicators have been adapted and expanded on for potential application in public PHC facilities in South Africa (51).

Here we aim to compare the model AWaRe-based primary care quality indicators developed at a global level with those nationally adapted for the South African public PHC sector as the first exemplar LMICs (50, 51). This paper also aims to examine the key factors affecting the appropriateness and feasibility of these indicators in the South African public PHC context. The findings can be used to help improve future antibiotic prescribing, not only in South Africa, but also in other LMICs.

## 2 Methodology

### 2.1 Study design and setting

We conducted a comparative analysis of two sets of AWaRe based quality indicators developed to assess the appropriateness and feasibility of antibiotic prescribing in primary care: (1) a set of model global indicators developed using both the Delphi technique and the RAM (49, 50), and (2) a set of model indicators adapted for public PHC settings in South Africa (51). This comparison focuses on public sector PHC as this is where most patients with common infections are currently treated in South Africa (22, 27, 52).

Full details of the development of the sets of indicators are provided elsewhere (50, 51). In brief, the global indicators were developed using both the Delphi technique and the Research and Development/University of California Los Angeles (RAND/UCLA) Appropriateness Method (RAM), while the South African indicators were developed using the RAM (49, 51, 53–56). The second round of the global RAM was conducted by a panel of 12 global experts in AMR/AMS while the second round of the South African RAM comprised a panel of 10 national multidisciplinary experts (49, 51). The second round of the global RAM rated 62 primary care indicators. In the second round of the South African RAM, 89 indicators were rated, including the 62 primary care indicators adapted from the global RAM (51). An overview of the methods used to develop both sets of indicators is presented in Supplementary Table S3.

### 2.2 Comparison of quality indicators

For this analysis, we focused on a subset of the indicators from each study: 62 overlapping indicators i.e. only indicators applicable to primary care settings and only those rated in the second RAM round of both sets. We compared the appropriateness and feasibility ratings of the global expert panel with the ratings of the South African expert panel, specifically the median appropriateness and feasibility ratings, and the level of consensus of agreement, equivocation and disagreement of these ratings (53, 54). The definitions and criteria used to determine the three levels of consensus of agreement, equivocation and disagreement for both indicator sets are presented in Supplementary Table S4.

### 2.3 Ethical approval

Ethical approval for the study was granted by the Sefako Makgatho University Research Ethics Committee (Ref: SMUREC/P/60/2025:PG). The study was conducted according to the methodology outlined in the study protocol approved by the committee on 13 February 2025. The study was also registered on the National Department of Health's National Health Research Database (Ref: GP_202508_096).

## 3 Results

Table 1 presents a summary of the median scores, i.e., scores from 1 to 9, and level of consensus, i.e., agreement [A], equivocation [E] or disagreement [D], for the 62 overlapping indicators rated for appropriateness and feasibility in the second rounds of the global RAM and the South African RAM.

**Table 1:** Round 2 median ratings and level of consensus for indicators rated by the global and the South African panels in the RAM second round (*n* = 62).

RAM indicators			RAM appropriateness ratings				RAM feasibility ratings			
			Global panel (*n* = 12)		South African panel (*n* = 10)		Global panel (*n* = 12)		South African panel (*n* = 10)	
Infection (*n*)	No.	Indicator statements	Median^1^ (LoC)^2^	% of panellists^3^	Median^1^ (LoC)^2^	% of panellists^3^	Median^1^ (LoC)^2^	% of panellists^3^	Median^1^ (LoC)^2^	% of panellists^3^
**RTI (*****n*** **=** **12)**	1	Proportion of all patients presenting with any acute RTI given an oral antibiotic	8 (A)	83.3	9 (E)	60	8 (E)	66.7	8 (A)	100
	2	Proportion of all patients presenting with any acute RTI given oral amoxicillin	8 (A)	91.7	8 (A)	100	8 (E)	75	8 (A)	100
	3	Proportion of all patients presenting with any acute RTI given any oral Access antibiotic (including amoxicillin)	8 (A)	91.7	8 (A)	100	8 (E)	75	8 (A)	100
	4	Proportion of all patients presenting with any acute RTI given any oral Watch antibiotic	8 (A)	91.7	8 (A)	100	7 (A)	91.7	7 (A)	100
	5	Proportion of patients presenting with bronchitis given an oral antibiotic	7 (E)	75	8 (A)	100	7 (A)	83.3	7 (E)	50
	6	Proportion of patients with any ear/sinus/throat infection (not pneumonia) given an oral antibiotic	8 (A)	83.3	8 (A)	100	8 (A)	83.3	8 (A)	100
	7	Proportion of patients with any ear/sinus/throat infection (not pneumonia) at high risk^*^ of severe complications given an oral antibiotic	8 (A)	100	8 (A)	100	8 (E)	66.7	8 (E)	70
	8	Proportion of patients with any ear/sinus/throat infection (not pneumonia) at high risk^*^ of severe complications given amoxicillin	7 (A)	91.7	8 (A)	100	7 (E)	75	8 (A)	80
	9	Proportion of patients with any ear/sinus/throat infection (not pneumonia) at high risk^*^ of severe complications given any oral Access antibiotic (including amoxicillin)	7 (A)	91.7	8 (A)	100	7 (A)	91.7	8 (A)	100
	10	Proportion of patients with acute RTIs given the duration in days of oral antibiotics recommended in the WHO AWaRe Antibiotic Book	7 (A)	83.3	8 (A)	100	6 (E)	50	8 (A)	100
	11	Proportion of patients with acute RTIs prescribed the total daily dose of oral antibiotics recommended in the WHO AWaRe Antibiotic Book	7 (E)	75	9 (E)	60	6 (E)	66.7	7 (A)	90
	12	Proportion of patients with bacterial RTIs given oral Access or Watch antibiotics	7 (E)	75	8 (A)	100	6 (E)	41.7	7 (A)	90
**Lower UTI (*****n*** **=** **7)**	13^**#**^	Proportion of patients presenting with lower UTI given an oral antibiotic	8 (A)	91.7	8 (A)	80	7 (A)	83.3	8 (A)	100
	14^**#**^	Proportion of low-risk^*^ patients with positive urine test (positive urine leucocytes/leucocyte esterase or positive urine culture), but no UTI symptoms (e.g., no dysuria, no increased urinary urgency and frequency, no lower abdominal pain or discomfort or sometimes visible haematuria), given oral antibiotics	7 (E)	66.7	8 (E)	70	5 (E)	50	8 (E)	70
	15^**#**^	Proportion of patients presenting with lower UTI given any oral Access antibiotic	8 (A)	91.7	8 (A)	80	7 (A)	83.3	8 (A)	100
	16	Proportion of patients with lower UTI given oral Watch antibiotics	7 (E)	75	8 (A)	90	7 (E)	75	8 (A)	100
	17	Proportion of patients presenting with lower UTI given any oral Watch antibiotic	8 (A)	91.7	8 (A)	90	7 (E)	75	8 (A)	90
	18^**#**^	Proportion of patients presenting with lower UTIs given the duration in days of oral antibiotics recommended in the WHO AWaRe Antibiotic Book	7 (A)	83.3	9 (E)	60	5 (E)	58.3	8 (A)	100
	19	Proportion of patients presenting with lower UTI prescribed the total daily dose of oral antibiotics recommended in the WHO AWaRe Antibiotic Book	7 (A)	100	8 (A)	90	6 (E)	50	8 (A)	100
**SSTI (*****n*** **=** **13)**	20	Proportion of patients presenting with acute lymphadenitis given an oral antibiotic	7 (E)	75.0	8 (A)	100	6 (E)	66.7	8 (A)	90
	21	Proportion of patients presenting with higher risk^*^ (of bacterial infection) acute lymphadenitis given an oral antibiotic	7 (A)	83.3	9 (E)	60	6 (E)	75	7 (A)	90
	22	Proportion of patients presenting with higher risk^*^ (of bacterial infection) acute lymphadenitis given any oral Access antibiotic	7 (A)	91.7	9 (A)	80	6 (E)	66.7	7 (A)	90
	23	Proportion of patients presenting with higher risk^*^ (of bacterial infection) acute lymphadenitis given any oral Watch antibiotic	7 (A)	83.3	8 (A)	100	6 (E)	58.3	7 (A)	80
	24	Proportion of patients with lower risk (of bacterial infection) acute lymphadenitis given oral antibiotics	7 (E)	75	8 (A)	90	5 (E)	75	7 (A)	90
	25	Proportion of patients presenting with acute lymphadenitis given the duration in days of oral antibiotics recommended in the WHO AWaRe Antibiotic Book	7 (E)	75	9 (A)	90	5 (E)	58.3	8 (A)	80
	26	Proportion of patients presenting with acute lymphadenitis prescribed the total daily dose of oral antibiotics recommended in the WHO AWaRe Antibiotic Book	6 (E)	66.7	9 (A)	90	5 (E)	75	8 (A)	80
	27	Proportion of patients presenting with mild^**^ SSTIs given an oral antibiotic	7 (A)	100	8 (A)	100	7 (A)	83.3	7 (A)	90
	28	Proportion of patients presenting with mild^**^ SSTIs given a topical antibiotic	7 (A)	83.3	8 (A)	90	7 (A)	83.3	8 (A)	90
	29	Proportion of patients presenting with mild^**^ SSTIs given any oral Access antibiotic	7 (A)	91.7	8 (A)	90	6 (E)	75	7 (A)	90
	30	Proportion of patients presenting with mild^**^ SSTIs given any oral Watch antibiotic	7 (A)	91.7	9 (E)	70	6 (E)	75	7 (A)	90
	31	Proportion of patients presenting with mild^**^ SSTIs given the duration in days of oral antibiotics recommended in the WHO AWaRe Antibiotic Book	7 (A)	100	9 (A)	90	5 (E)	66.7	8 (A)	90
	32	Proportion of patients presenting with mild^**^ SSTIs prescribed the total daily dose of oral antibiotics recommended in the WHO AWaRe Antibiotic Book	7 (A)	100	8 (A)	90	6 (E)	58.3	8 (A)	90
**Diarrhea and enteric fever (*****n*** **=** **9)**	33	Proportion of all patients presenting with high-risk^*^ acute infectious non-bloody diarrhea given any oral Watch antibiotic	7 (A)	83.3	8 (A)	100	6 (E)	50	7 (A)	90
	34	Proportion of all patients presenting with acute infectious non-bloody diarrhea given oral antibiotics	8 (E)	66.7	9 (E)	70	7 (A)	83.3	8 (E)	70
	35	Proportion of all patients presenting with high-risk^*^ acute infectious non-bloody diarrhea given any oral Access antibiotic	8 (A)	83.3	9 (E)	70	7 (E)	58.3	7 (A)	90
	36	Proportion of otherwise healthy patients presenting with acute infectious non-bloody diarrhea given an oral antibiotic	7 (E)	75.0	9 (A)	80	6 (E)	66.7	7 (A)	90
	37	Proportion of patients presenting with high-risk^*^ acute infectious non-bloody diarrhea given the duration in days of oral antibiotics recommended in the WHO AWaRe Antibiotic Book	7 (A)	83.3	8 (A)	90	4 (E)	75	7 (A)	90
	38	Proportion of patients presenting with high-risk^*^ acute infectious non-bloody diarrhea prescribed the total daily dose of oral antibiotics recommended in the WHO AWaRe Antibiotic Book	7 (E)	75.0	8 (A)	90	5 (E)	66.7	7 (A)	90
	39	Proportion of patients with acute bloody infectious diarrhea given oral Access or Watch antibiotics	7 (E)	75	8 (A)	100	7 (E)	75	8 (A)	80
	40	Proportion of patients with acute bloody infectious diarrhea given oral Access antibiotics	8 (A)	83.3	9 (A)	90	7 (E)	75	8 (E)	70
	41	Proportion of patients with acute bloody infectious diarrhea given oral Watch antibiotics	8 (A)	100	9 (A)	80	7 (E)	66.7	8 (E)	70
**Bacterial eye (*****n*** **=** **6)**	42	Proportion of patients presenting with eye infection given an oral antibiotic	8 (A)	83.3	8 (E)	70	8 (E)	75	7 (E)	60
	43	Proportion of patients presenting with bacterial eye infections given the duration in days of oral antibiotics recommended in the WHO AWaRe Antibiotic Book	7 (E)	58.3	8 (A)	100	4 (E)	41.7	8 (A)	90
	44	Proportion of patients with bacterial eye infections prescribed the total daily dose of oral antibiotics recommended in the WHO AWaRe Antibiotic Book	6 (E)	58.3	8 (A)	100	4 (E)	50	8 (A)	90
	45	Proportion of patients with eye infections prescribed the total daily dose of oral antibiotics recommended in the WHO AWaRe Antibiotic Book	5 (D)	33.3	7 (A)	90	5 (E)	58.3	8 (A)	90
	46	Proportion of patients presenting with eye infection at high risk^*^ of severe complications given any oral Access antibiotic	7 (A)	100	9 (E)	60	6 (E)	75	7 (A)	90
	47	Proportion of patients presenting with eye infection at high risk^*^ of severe complications given any oral Watch antibiotic	7 (A)	91.7	9 (E)	70	6 (E)	75	7 (A)	90
**Dental (*****n*** **=** **9)**	48	Proportion of otherwise healthy adults presenting with dental infections given an oral antibiotic	8 (E)	75	8 (A)	100	6 (E)	41.7	8 (A)	90
	49	Proportion of otherwise healthy adults presenting with severe^**^ dental infections given an oral antibiotic	8 (A)	83.3	9 (A)	90	6 (E)	41.7	8 (A)	90
	50	Proportion of patients presenting with dental infections at high risk^*^ of severe complications given an oral antibiotic	8 (E)	75	8 (A)	100	5 (E)	58.3	7 (A)	90
	51	Proportion of patients presenting with dental infections at high risk^*^ of severe complications given amoxicillin	7 (A)	83.3	8 (A)	100	6 (E)	50	7 (A)	90
	52	Proportion of patients presenting with dental infections at high risk^*^ of severe complications given any oral Access antibiotic (including amoxicillin)	8 (E)	75	8 (A)	90	6 (E)	50	7 (A)	80
	53	Proportion of patients with lower risk (of bacterial infection) dental infections given oral antibiotics	7 (A)	91.7	8 (A)	100	6 (E)	25	7 (A)	90
	54	Proportion of patients presenting with dental infections given the duration in days of oral antibiotics recommended in the WHO AWaRe Antibiotic Book	7 (A)	83.3	8 (A)	90	5 (E)	50	8 (A)	100
	55	Proportion of patients with dental infections prescribed the total daily dose of oral antibiotic recommended in the WHO AWaRe Antibiotic Book	7 (E)	75	8 (A)	100	6 (E)	58.3	8 (A)	100
	56	Proportion of patients with dental infections given oral Watch antibiotics	7 (A)	83.3	8 (A)	100	7 (E)	75	8 (A)	100
**General (*****n*** **=** **6)**	57^**#**^	Allergy status of the patient including timing, nature and severity of previous exposure/possible allergic reactions to antibiotics and the name/s of the antibiotic should be documented in the medical records	8 (A)	100	9 (A)	90	6 (E)	58.3	5 (E)	50
	58	Any toxicity/adverse reaction to an antibiotic (including type, duration of symptoms, time of onset since antibiotic administration) should be documented in medical records	8 (A)	91.7	9 (A)	100	5 (E)	58.3	5 (E)	40
	59	Use of oral Access and Watch antibiotics (split by AWaRe group) measured in DID in primary care	8 (A)	83.3	9 (A)	90	6 (E)	66.7	7 (E)	70
	60	At least 80% of total oral antibiotic use in primary care should be Access antibiotics	8 (E)	75	9 (A)	90	6 (E)	58.3	8 (A)	100
	61	Percentage of total oral Access antibiotic use	8 (A)	100	9 (A)	90	8 (E)	75	8 (A)	90
	62^**#**^	Ratio of oral amoxicillin measured in DID in primary care to all oral antibiotics in primary care measured in DID excluding amoxicillin (including amoxicillin-clavulanic acid)	7 (A)	83.3	8 (A)	100	6 (A)	83.3	8 (A)	90

NB: ^**#**^Indicators rephrased for the South African RAM (see Table 2 for details on the rephrased statements);

^1^Panellist median rating on a scale of 1–9;

^2^LoC, level of consensus (A, Agreement; E, Equivocal; D, Disagreement);

^3^Percentage of panelists that rated within the three-point scale of the median (within +/−1 of the median);

DID, defined daily doses per 1,000 inhabitants per day; RTI, respiratory tract infection; SSTI, skin and soft tissue infection; UTI, lower urinary tract infection;

^*^risk criteria as per AWaRe guidance (44, 45);

^**^severity criteria as per AWaRe guidance (44, 45).

Overall, the median ratings and level of consensus for the 62 overlapping indicators were higher for the South African RAM than the global RAM for all infection and general categories. For appropriateness ratings, no indicators from the global RAM had a median score of 9, 23 indicators (37.1%) had a median score of 8, 36 indicators (58.1%) had a median score of 7, two indicators (3.2%) had a median score of 6 and 1 (1.6%) indicator had a median score of 5. For the global RAM, the highest median score for appropriateness was 8, and indicators with this score were mostly general indicators (5/6; 83.3%) and respiratory tract infections (RTIs; 6/12; 50%). For the South African RAM, 22 indicators (35.5%) had a median score of 9 for appropriateness, 39 indicators (62.9%) scored 8 and one indicator (1.6%) scored 7. For the South African RAM, the highest median score for appropriateness was 9 and indicators with this score were mostly general indicators (5/6; 83.3%) and diarrhea and enteric fever (5/9; 55.6%). The lowest median score for appropriateness was 5 for the global RAM and 7 for the South African RAM and was in the bacterial eye infections category (1/6; 16.7%) for both sets.

For feasibility ratings, no indicators from the global RAM had a median score of 9, seven indicators (11.3%) scored 8, 16 indicators (25.8%) scored 7, 25 indicators (40.3%) scored 6, 11 indicators (17.7%) scored 5 and three indicators (4.8%) scored 4. For the South African RAM, no indicators had a median score of 9, 36 indicators (58.1%) scored 8, 24 indicators (38.7%) scored 7 and two indicators (3.2%) scored 5. For the global and South Africa RAM, the highest median score for feasibility was 8. For the global RAM, the highest median score was mostly achieved for RTIs (5/12; 41.7%). For the South African RAM, the highest median score was for lower urinary tract infections (UTIs; *n* = 7; 100%) and RTIs (8/12; 66.7%). For the global RAM, the lowest feasibility score was 4, which was for bacterial eye infections (2/6; 33.3%) and diarrhea and enteric fever (1/9; 11.1%). For the South African RAM, the lowest feasibility score was 5, which was for general category indicators (2/6; 33.3%).

Nine global RAM indicators (14.5%) scored 100% for level of consensus among panelists for appropriateness, while 25 indicators (40.3%) scored 100% for the South African RAM. Two identical indicators scored 100% for level of consensus by both sets of panelists for appropriateness (indicators seven and 27; Table 1). For the global RAM, the indicators with the highest level of consensus for appropriateness were general category (5/6; 83.3%) and RTI (9/12; 75%) indicators. For the South African RAM, the indicators with the highest level of consensus for appropriateness were general category (*n* = 6; 100%) and dental infection (*n* = 9; 100%) indicators. For both the global and the South African RAM, the indicators with the lowest level of consensus for appropriateness were bacterial eye infection indicators (3/6; 50%). No indicators scored 100% for level of consensus among the global RAM panelists for feasibility, while 16 indicators (25.8%) scored 100% for the South African RAM. For the global RAM, the indicators with the highest level of consensus for feasibility were RTIs (4/12; 33.3%) and lower UTIs (2/7; 28.6%). For the global RAM, the indicators with the lowest level of consensus for feasibility were bacterial eye infections (0%) and dental infections (0%). For the South African RAM, the indicators with the highest level of consensus for feasibility were dental infections (*n* = 9; 100%) and skin and soft tissue infections (SSTIs; *n* = 13; 100%). For the South Africa RAM, the indicators with the lowest level of consensus for feasibility were general category indicators (3/6; 50%).

For the 62 overlapping indicators, 59 indicators (95.2%) were rated appropriate for the global RAM while all indicators were rated appropriate (100%) for the South African RAM. Forty-one indicators (66.1%) were rated appropriate with agreement for the global RAM, and 51 indicators (82.3%) were rated appropriate with agreement for the South African RAM. Eight indicators had identical ratings and level of consensus for appropriateness between the two sets (indicators 2–4, 6, 7, 13, 15 and 17; Table 1) with a rating score and level of consensus of 8A. Thirty-three identical indicators (53.2%) were rated appropriate with agreement for both sets. Of these, eight indicators (24.2%) were for RTIs, seven indicators (21.2%) were for SSTIs, four indicators (12.1%) were for lower UTIs, four indicators (12.1%) were for diarrhea and enteric fever, five indicators (15.2%) were for dental infections and five indicators (15.2%) were general indicators.

For the global RAM ratings, 23 indicators (37.1%) were rated feasible while 60 indicators (96.8%) were rated feasible for the South African RAM. Nine indicators (14.5%) were rated feasible with agreement for the global RAM, and 52 indicators (83.9%) were rated feasible with agreement for the South African RAM. Five indicators had identical median ratings and level of consensus for feasibility between the two sets, of these three indicators were in agreement and two were equivocal [indicators 4 (7A), 6 (8A), 7 (8E), 27 (7A) and 58 (5E); Table 1].

Seven indicators (11.3%) were rated both appropriate with agreement and feasible with agreement by the global RAM panelists. Of these, three indicators were for RTIs, and two indicators each for SSTIs and UTIs. Forty-six indicators (74.2%) were rated both appropriate with agreement and feasible with agreement by the South African panelists. Of these, eight indicators (17.4%) were for RTIs, six indicators (13%) were for lower UTIs, 11 indicators (23.9%) were for SSTIs, five indicators (10.9%) were for diarrhea and enteric fever, three indicators (6.5%) were for bacterial eye infections, nine indicators (19.6%) were for dental infections and four (8.7%) were general indicators. The same seven indicators (11.3%) rated both appropriate and feasible with agreement by the global RAM are the only identical indicators rated both appropriate and feasible with agreement for both sets.

There was equivocation for both the rating scores and level of consensus for appropriateness for two indicators for the global RAM, with a median rating score and level of consensus of 6E (indicators 26 and 44; Table 1). There was equivocation for both the rating scores and level of consensus for feasibility for 38 indicators for the global RAM, with a median rating score and level of consensus of 6E (*n* = 24), 5E (*n* = 11) and 4E (*n* = 3), as shown in Table 1. There was equivocation for both the rating scores and level of consensus for feasibility for two indicators for the South African RAM, with a median rating score and level of consensus of 6E. For the global and South African RAM, no indicator had a median score of 1–3 for both appropriateness and feasibility. There was one indicator for the global set rated equivocal with disagreement for appropriateness, with a median score and level of consensus of 5D (indicator 45; Table 1). There was no disagreement for feasibility ratings for the global RAM. For the South African RAM, there was no disagreement (≥33% rated between 1 and 3 and ≥33% rated between 7 and 9) for both appropriateness and feasibility ratings.

### 3.1 Rephrased indicators for the South African context

Sixty-two indicators were compared of which 56 were identically worded between the two panels. Six of the 62 indicators were rephrased by the South African RAM panelists for the local South African context (indicators 13–15, 18, 57 and 62; Table 1). The word “oral” was removed from four UTI indicators (indicators 13–15 and 18; Table 1) to reflect the first line treatment recommended in the national standard treatment guidelines (STGs) for uncomplicated UTIs in South Africa, which includes gentamicin injection (51, 57). One general indicator (indicator 57; Table 1) was simplified to only record the allergy status of the patient because recording all the details pertaining to an antibiotic allergy in the patient file was considered infeasible for the current South African context. Another general indicator (indicator 62; Table 1) was rephrased to improve the clarity of the statement.

Five of the six rephrased indicators (indicators 13–15, 18 and 57; Table 1) from the global set, were rated both appropriate and feasible with agreement, by the South African RAM. The sixth indicator (indicator 62; Table 1) was rated both appropriate and feasible as the original statement. Two of the 6 original statements (indicators 13 and 15; Table 1) that were rephrased for South Africa were rated both appropriate and feasible with agreement by the global RAM panel. Details of the rephrased statements are shown in Table 2.

**Table 2:** Rephrased quality indicator statements from the model global indicators included in the final set for South Africa.

No.	Original statement	Rephrased statement
13	Proportion of patients presenting with lower UTI given an oral antibiotic	Proportion of patients presenting with lower UTI given an antibiotic
14	Proportion of low-risk^*^ patients with positive urine test (positive urine leucocytes/leucocyte esterase or positive urine culture), but no UTI symptoms (e.g., no dysuria, no increased urinary urgency and frequency, no lower abdominal pain or discomfort or sometimes visible haematuria), given oral antibiotics	Proportion of low-risk^*^ patients with positive urine test (positive urine leucocytes/leucocyte esterase or positive urine culture), but no UTI symptoms (e.g., no dysuria, no increased urinary urgency and frequency, no lower abdominal pain or discomfort or sometimes visible haematuria), given antibiotics
15	Proportion of patients presenting with lower UTI given any oral Access antibiotic	Proportion of patients presenting with lower UTI given any Access antibiotic
18	Proportion of patients presenting with lower UTIs given the duration in days of oral antibiotics recommended in the WHO AWaRe antibiotic book	Proportion of patients presenting with lower UTIs given the duration in days of antibiotics recommended in the WHO AWaRe antibiotic book
57	Allergy status of the patient including timing, nature and severity of previous exposure/possible allergic reactions to antibiotics and the name/s of the antibiotic should be documented in the medical records	Allergy status of the patient to any antibiotic should be documented in the medical records
62	Ratio of oral amoxicillin measured in DID in primary care to all oral antibiotics in primary care measured in DID excluding amoxicillin (including amoxicillin-clavulanic acid)	Ratio of oral amoxicillin measured in DID in primary care to all other oral antibiotics in primary care measured in DID excluding amoxicillin (including amoxicillin-clavulanic acid)

NB: Indicator number refers to Table 1;

DID, defined daily doses per 1,000 inhabitants per day; UTI, urinary tract infection;

^*^risk criteria as per AWaRe guidance (44, 45).

### 3.2 Additional quality indicators developed for the South African context

In addition to the overlapping indicators compared in this study and the rephrased indicators, 15 additional indicators for sexually transmitted infections (STIs) and acute cough were developed specifically for the South African context, and two new indicators for bacterial eye infections were added by the South African panelists during the RAM consensus meeting (51).

## 4 Discussion

We believe this is the first study to assess differences between global model AWaRe-based antibiotic prescribing quality indicators with those nationally adapted for the South African public PHC context. While the AWaRe-based model indicators may be globally applicable, they are still context sensitive and need to be adapted before implementation in different countries (50, 58–60). Our study allowed the evaluation of the transferability and relevance of the global model indicators within the South African public PHC context. This is critical given appreciable differences in antibiotic utilization rates, AMR profiles, and AMS activities between high-income countries and LMICs (1, 11, 21, 26, 31). Of the 62 indicators we compared, all the indicators were rated appropriate for the South African RAM, and most indicators (95.2%) were rated appropriate for the global RAM. The high appropriateness ratings for both sets suggest that most indicators are considered beneficial, evidence-based, or clinically indicated, when applied to the average patient in PHC settings both in the South African and global contexts. Sixty indicators were rated feasible by the South African RAM compared to 23 for the global RAM. These results suggest that while most indicators are currently not feasible for most PHC settings globally, they can currently be implemented in routine clinical practice in the South African context. This could also be because of more varied primary care systems, contextual differences in documentation in primary care, different PHC structures and differing concerns regarding implementation of indicators for different PHC contexts represented by the ratings from the global panel.

The adaptation process for South Africa resulted in rephrasing only six (9.7%) indicators to suit the local context and align with the first line treatment recommended in the national treatment guidelines (57). However, most of the indicators from the global model set were not rephrased, which highlights the robustness of the processes used to develop the model indicators for different national contexts, using guidance from the WHO AWaRe antibiotic book (44, 46, 50). These results also show that the South African national STGs are largely aligned with the AWaRe guidance for the management of common infections treated in primary care (44, 46, 57).

Only 7 identical indicators were rated both appropriate and feasible with agreement by both the global and South African panelists, which means that while other indicators might have high scores for appropriateness or feasibility by median score (rated between 7 and 9), there wasn't sufficient agreement among the panelists regarding that score. The South African RAM had a higher proportion of indicators rated both appropriate and feasible with agreement compared to the global set, reflecting a higher level of consensus, which strengthens confidence that the indicators are locally acceptable, appropriate and applicable. These results emphasize the importance of local adaptation of model global indicators to the South African context, so that the indicators reflect global evidence and can be applied locally. These results also suggest that the indicator set for South Africa was considered clinically sound and contextually realistic by the panelists, with strong potential for practical use in evaluating and improving antibiotic prescribing quality in South African PHC facilities (21, 27). This is likely because South Africa has a relatively strong public PHC system, with an established PHC clinic network, strong policy commitment to strengthening PHC, strong public health programme experience in high-burden diseases, and established district and provincial governance structures (61–63). These factors support the application of the indicators consistently across many similarly structured facilities, using existing data collection tools and systems such as routine clinical audits. In addition, the data required to apply the indicators is available from patient files and is routinely documented during patient consultations, as demonstrated by a recent point prevalence study conducted at PHC facilities to assess antibiotic prescribing patterns (64). Public PHC facilities already conduct routine clinical audits, for example, the Ideal Clinic audits, which supports the integration of the quality indicators into these audits and there is existing expertise to successfully implement the quality indicators in practice (65).

The South African STGs for PHC are largely aligned with the WHO AWaRe guidance, which contributes to the feasibility of the indicators and is important for the successful adaptation and implementation of the quality indicators, given concerns with the robustness of national antibiotic guidelines among other LMICs (44, 46, 48, 57). The regular revision and adherence to national guidelines, aligned with the WHO AWaRe framework, can appreciably strengthen AMS efforts to reduce AMR (47, 59, 66). The national guidelines for South Africa are regularly updated, which is important to consider prevailing resistance rates and ensure that quality indicators that assess adherence to guidelines are meaningfully interpreted (48, 57, 59, 67).

While these indicators were given high feasibility ratings by the South African RAM, the practical implementation and measurement of the indicators should be carefully considered in practice. This is because incomplete documentation and missing diagnoses could result in fewer indicators being measurable or in misclassifying appropriate prescribing as inappropriate or vice versa, particularly for those indicators that are disease-specific or diagnosis-based (60). The reliance on paper-based records in the public health system in most provinces in South Africa may also present a barrier to the implementation of all the quality indicators at once (68–70). Consequently, a phased implementation approach and integrating priority quality indicators, for example RTI indicators, into existing routine quality audits facilitated by districts, is recommended to improve uptake and feasibility, without overburdening available human resources. In the long term, improvement of electronic prescribing systems and investment in AMS teams in PHC facilities may help uptake of quality indicators in public PHC facilities in South Africa. An important contextual challenge that should be considered for South Africa is supply chain constraints, which can result in shortages and stock-outs of antibiotics, including Access antibiotics, resulting in high Watch proportions (71–74). While indicators are appropriate, feasible and measurable, actual percentage scores of the indicators may be low. As a result, supply chain constraints may be interpreted as inappropriate prescribing, highlighting the importance of strengthening antibiotic supply chains and their availability to strengthen AMS in PHC settings (75).

Fewer indicators being rated as feasible in the global RAM suggest feasibility gaps across different countries globally, especially among LMICs with more limited resources. Adapting model indicators to any given country, with concurrent local implementation strategies, is important because appropriateness and feasibility are context dependent. This is because there can be potential differences in healthcare infrastructure and patient care-seeking as well as in national antibiotic treatment guidelines between countries, prescribing cultures, epidemiology and infectious disease burden (21, 41, 46, 49, 76). In addition, differences in diagnostic capacity, resistance patterns, data infrastructure, antibiotic availability, AMS policies, measurement units and denominators, as well as variation in resources and workforce expertise, affecting appropriateness and feasibility (21, 36, 41, 76–78). As the global RAM panel included experts representing a wide range of contexts, the low feasibility ratings and lack of consensus highlights the broad differences in PHC globally and the difficulty that may arise in implementing global model indicators in different contexts. Local adaptation and prioritization will be essential for other countries to consider when seeking to develop and implement appropriate quality indicators. In the South African context, the indicator adaptation process also resulted in the development of additional indicators for STIs and acute coughs as these are common infection presentations managed in public PHC facilities in South Africa which were not covered by the global indicators (51). Similarly, other countries may have important infections in their settings that are not included in the global model indicators that should be included in locally adapted sets.

We believe a strength of this study is the comparative assessment of globally developed model quality indicators with nationally adapted indicators to assess contextual adaptation and identify factors affecting implementation. Both sets of indicators used the same consensus methodology, definitions for consensus and data analysis methods, for improved comparison. The indicators from both sets covered a wide range of common infections seen and treated in PHC settings, across six infection categories and one general category (49, 51). The final set of indicators for South Africa includes an additional category for STIs, which were developed specifically for South Africa, highlighting the value of the adaptation process (51). This is favorable as these eight categories reflect the current infectious disease symptoms that patients currently present with at public PHC facilities in South Africa (22, 64, 79). Consequently, the indicators are focused on the key prescribing quality issues in the South African public PHC sector. This comprehensive national set can serve as model indicators for different provinces or districts in South Africa, to improve antibiotic prescribing and potentially assess prescribing practices between facilities. This is important considering there are currently limited published indicators that have been used in South African primary care, with no recent published evidence that these indicators are still in use and have led to improved antibiotic prescribing, as shown in Supplementary Table S2.

We are aware that the study had some limitations. Both sets of indicators are model indicators and may fail to account for all implementation realities and prescribing complexities applicable to different PHC facilities in South Africa and other countries. The two studies also focused on the average patient and may potentially overlook other detailed clinical information, potentially limiting the precision of quality measurement for both global and national indicators. There was careful selection of diverse and multidisciplinary panelists across all respective panels and panel sizes accorded with recommendations. However, while the RAM aims to systematically manage subjective judgement through evidence summaries, guidance documents and structured deliberation, subjective interpretation and consensus bias cannot be fully eliminated. Four of the South African RAM panelists participated in the global Delphi panel, which may have unintentionally contributed to the high ratings and support for indicators previously endorsed in the global Delphi process. This may have possibly contributed to the fewer differences in indicator phrasing and relevance for South Africa than might occur in another country that was not involved in the global RAM. The co-chair for the South African consensus meeting (MM) participated in the global RAM, however he did not participate in the rating process for South Africa. Despite these limitations, we believe both sets of indicators are robust and evidence-based and serve as valuable tools for AMS in primary care.

The clinimetric properties of the indicators developed for South Africa need to be tested in clinical practice. Ongoing research, building on this study, will be focused on testing the applicability and measuring the clinimetric properties of the quality indicators in selected South African public PHC clinics. This is an important step, and already happening (80), and is a key part of prioritization of indicators before widespread implementation of agreed indicators within public PHC facilities in South Africa. We will continue to build on our findings to improve future antibiotic prescribing in PHC facilities in South Africa.

## 5 Conclusion

The final set of quality indicators for the South African public PHC sector represents the first set of nationally adapted AWaRe-based quality indicators from a global model set to assess the appropriateness of antibiotic prescribing. Most indicators from this national set were adapted from the model global set and were rated both appropriate and feasible for the local context. This study illustrates that the model global indicators serve as a good starting point for adapting quality indicators at a national level and South Africa panelists considered most of the indicators to be feasible in PHC compared to a global panel. We believe the findings from this study can serve as a benchmark for other LMICs intending to adapt the model indicators for their countries.

## Data Availability

The original contributions presented in the study are included in the article/Supplementary material, further inquiries can be directed to the corresponding authors.
