# Supplementary material for: Comparison between model global AWaRe-based quality indicators and quality indicators developed to assess the appropriateness of antibiotic prescribing in primary healthcare in South Africa
Source: Front Public Health. 2026 Jul 3;14:1847084. doi: 10.3389/fpubh.2026.1847084 (PMC13376263; doi:10.3389/fpubh.2026.1847084)
Supplement: Supplementary file 1 [file Supplementary_File_1.pdf]

Supplementary Tables

Supplementary Table S1 – Current antibiotic prescribing concerns in primary care in South Africa

Author and year	Aim and methods	Summary of key findings
Farley et al., 2018	• Cross sectional survey design with a self-administered questionnaire to assess attitudes, knowledge and practices regarding antibiotics and ABR among primary care prescribers<br>• 264 prescribers completed the survey, 98.3% were physicians and 84.8% were practising in the private sector	• 95.8% of prescribers viewed ABR as a major challenge in South Africa<br>• 87.5% of prescribers indicated a need for further education on the appropriate use of antibiotics in primary care, and 96.2% expressed a desire for access to local ABR data to guide future prescribing<br>• Prescribers were also interested in the provision of STGs in different formats to improve future prescribing<br>• However, 66.5% of prescribers reported feeling pressured by patients to prescribe antibiotics for their presenting infectious illness, regardless of whether the antibiotics were needed
Gasson et al., 2018	• Antibiotic prescribing habits were assessed retrospectively among prescribers in 8 PHCs alongside potential reasons for non-adherence to STGs<br>• 654 patient records were reviewed	• 68.7% of patients attending these PHCs were prescribed an antibiotic<br>• There were concerns with low adherence to STGs as adherence only occurred in 45.1% of prescriptions<br>• The main reasons for non-adherence to STGs included: undocumented diagnoses (30.5%), antibiotics not required including for self-limiting viral infections (21.6%), incorrect doses of antibiotics prescribed (12.9%), incorrect duration of antibiotic therapy prescribed (9.5%), and incorrect treatment for the presenting infectious disease (1.5%)
Truter et al., 2018	• Study used a self-administered questionnaire to determine antibiotic prescribing habits among primary care physicians<br>• 16 community pharmacists in the Eastern Cape province participated in the study	• 81.3% of participants believed physicians over-prescribed antibiotics, including for viral infections, influenced by patient pressure<br>• Amoxicillin/co-amoxiclav were the most prescribed antibiotics, followed by clarithromycin, ciprofloxacin and azithromycin<br>• Surveyed community pharmacists believed sinusitis and URTIs were the most common infections for which antibiotics were prescribed
van Hecke et al., 2019	• The objective was to determine the perceptions of clinicians working in PHCs concerning antibiotic prescribing for acute coughs and UTIs alongside their experiences concerning point-of-care testing<br>• Qualitative interviews were undertaken among 23 prescribers	• Antibiotic prescribing decisions among participating HCPs in PHCs were typically influenced by a number of factors, including their clinical assessment of presenting patients, their comorbidities and perceptions regarding patient expectations<br>• There were observed difficulties in the communication between prescribers and patients, which often hampered efforts to explain non-antibiotic management strategies to patients including for viral infections<br>• In view of this, participating clinicians were typically positive towards current and future point-of-care testing, especially for viral infections, to improve evidence-based antibiotic prescribing

Author and year	Aim and methods	Summary of key findings
		• Prescribers though had concerns with current resources and workflow issues influencing the uptake and use of such tests as part of their routine care of patients presenting with infectious diseases
Govender et al., 2021	• The study aimed to evaluate the use and implementation of the STGs/EML among 98 prescribers (nurses) at a public tertiary institution and associated PHC facilities<br>• A mixed approach was used. This included evaluating patient records and undertaking interviews using a structured questionnaire	• Only 41.8% of nurses had access to the latest STGs/EML<br>• All the participating nurses stated they often/ sometimes refer to the STGs/EML when managing patients.<br>• 78.3% of prescriptions had the diagnosis recorded, with a 59.7% adherence rate to the STGs/EML.<br>• Most nurses (94.9%) requested training on the use of STGs/EML to improve future antibiotic prescribing
Alabi and Essack, 2022	• The objective was to assess the appropriateness of antibiotic prescribing among practicing GPs in the private sector<br>• This included an analysis of antibiotic prescriptions (188,141) among 174,889 patients, with appropriateness based on ICD-10 classification and whether an antibiotic was deemed warranted or not	• 92.9% of patients were prescribed one antibiotic by primary care GPs when attending their clinics, with 7.1% prescribed two or more antibiotics<br>• Penicillins were the most prescribed antibiotics (40.7%) of all antibiotics prescribed. This was followed by macrolides (16.8%) and cephalosporins (15.7% - all generations combined)<br>• 46.1% of all diagnoses made included diseases of the respiratory system<br>• 8.8% of all the prescriptions were subsequently deemed as appropriate and 32.0% potentially appropriate. However, 45.4% of prescriptions were seen as inappropriate and 13.8% could not be assessed due to a lack of specific codes/ contained unlisted codes/ or contained unclear descriptions in the prescription
De Vries et al., 2022	• The objective was to evaluate the impact of a multidisciplinary audit and feedback AMS intervention to improve future antibiotic prescribing<br>• The AMS included monthly feedback meetings at 13 PHCs using 10 prescriptions randomly selected for peer review<br>• Antibiotic prescriptions were subsequently scored for adherence to seven key measures including antibiotic choices vs. recommendations in STGs<br>• Antibiotic utilization patterns were also assessed	• There was suboptimal adherence to the current STGs at the start of the study at only 11%<br>• Encouragingly, adherence increased to 53% over a 2-year period<br>• However, adherence to STGs was significantly lower in the winter and spring - concurrent with higher antibiotic prescribing/consumption. This potentially reflects inappropriate antibiotic prescribing for acute viral ARIs during these months<br>• Only 19% of prescriptions were correct in the first 6 months. This rose toa mean of 47% correct prescriptions in the last 6 months of the study ($p<0.001$) following active interventions.<br>• Overall, the AMS was associated with a 19.3% decrease in antibiotic consumption during the study period

Author and year	Aim and methods	Summary of key findings
Guma et al., 2022	• A semi-structured web-based questionnaire was used to assess current antibiotic empiric prescribing rates among 209 private GPs for their patients attending with ARIs and associated key factors	• 55.5% of surveyed GPs admitted to prescribing antibiotics empirically for patients with ARIs more than 70% of the time - primarily for symptom relief and the prevention of complications<br>• Encouragingly, GPs with more experience and working alone were slightly less likely to prescribe antibiotics empirically<br>• Key factors significantly associated with empiric prescribing of antibiotics were workload/time pressures, diagnostic uncertainty and the use of a formulary
Keuler et al., 2022	• The aim was to assess the treatment of UTIs in 6 PHCs and determine their compliance with current STGs/EML<br>• This was a retrospective review of 401 UTI episodes for 383 patients	• Antibiotics were prescribed in all male and 98.5% of females with uncomplicated UTIs and in 98.3% of those with complicated UTIs<br>• Nitrofurantoin was prescribed to most patients UTIs (57.1%), followed by ciprofloxacin (39.7%). Nitrofurantoin appropriately selected in 75.0% of patients with uncomplicated UTIs<br>• In patients with complicated cases, compliance to STGs/ EML was higher with ciprofloxacin (44.4%) vs. nitrofurantoin (25.6%)<br>• Overall compliance with STGs was greater for patients with uncomplicated (61.5%) vs. complicated UTIs (52.9%), with failure to comply with STGs mostly due to inappropriate antibiotic selection for complicated UTIs and the duration of prescribed antibiotics
Lagarde et al., 2023	• The objective was to assess prescribing practices for young and healthy SPs presenting to PHCs with viral bronchitis including both private (99 SPs) and public PHCs (102 SPs)<br>• Alongside this, 125 providers (across both sectors) were also interviewed face-to-face using a structured questionnaire	• Antibiotics were recommended in 72.6% of SP consultations, higher in the public sector (78.4%) vs. private sector (66.7%) - enhanced by perceived patient pressure<br>• These high rates were despite 84% of prescribers knowing the SP case was likely to be a viral infection (88% in the private sector vs. 77% in the public sector) and 58% knowing that antibiotics would not hasten recovery (40% public vs. 68% private)<br>• 47% of prescribers in public PHCs thought patients would not come back if they did not prescribe an antibiotic – higher in the private sector at 72% - despite SPs not demanding antibiotics<br>• Encouragingly, antibiotic prescribing rates were lower in both sectors (20% lower) in a previous study when HCPs were explicitly told by their patients that they did not want antibiotics for their infection unless they were necessary
Wieters et al., 2024	• The aim was to assess self-reported antibiotic use among 19 700 patients visiting healthcare facilities with acute RTI, acute gastrointestinal infection (GI) and acute febrile disease of unknown cause (AFDUC)<br>• The study was conducted in 4 African countries (Côte d'Ivoire, Burkina Faso, Democratic Republic of Congo, and South Africa)	• Out of the 7 258 (36.8%) patients who had taken antibiotics in the previous 10 days, 41.5% were prescribed for RTIs, 30.3% for AFDUC and 22.6% for GI infections. There were similar rates for RTIs in South Africa at 41.4% and AFDUC at 27.8%<br>• The most common antibiotic prescribed in the study was ceftriaxone (31.7% of antibiotics prescribed – lower in South Africa)<br>• Among patients with RTIs, the prescribing of ampicillin was highest in South Africa (22.8%) – with ceftriaxone at 15%

Author and year	Aim and methods	Summary of key findings
Chigome et al., 2025	• Multiple PPS were conducted among PHCs in two provinces in South Africa, as part of a larger global study	• Data for 615 patients were recorded in the PPS study with the most common symptoms for antibiotics being a genital discharge (21.8%), painful urination (18.4%), acute cough (17.7%), and a sore throat (13.5%), with patients potentially having more than one symptom<br>• At least one antibiotic was prescribed for 87% of attending patients<br>• Access antibiotics accounted for 53.4% of antibiotics prescribed, with 46.6% being from the Watch group. Ceftriaxone (29.7%), amoxicillin (29.4%) and azithromycin (28.4%) were the most prescribed antibiotics<br>• Overall, there are considerable concerns with current prescribing practices among PHCs in South Africa
Maluleke et al., 2025	• Study aimed to assess the extent of antibiotic purchasing without a prescription among independent, chain and franchise community pharmacies in a rural province<br>• A questionnaire was used to collect data from community pharmacists and pharmacist assistants<br>• 128 pharmacies participated and 313 questionnaires were completed by 106 pharmacists and 207 pharmacist assistants	• Participants from 88 (68.8%) pharmacies reported dispensing antibiotics without a prescription during the 14 days preceding the survey<br>• Antibiotics accounted for 47.9% of all medicines dispensed in the surveyed community pharmacies with penicillins the most dispensed (41.1%). 47.2% of antibiotics dispensed included cephalosporins, macrolides and fluoroquinolones<br>• STIs (33.5%) and URTIs (25.8%) were the most frequent indications for antibiotics<br>• 98.1% of community pharmacists and 97.6% of pharmacist assistants indicated they always or mostly offered symptomatic relief before suggesting/ dispensing antibiotics without a prescription to patients with typically self-limiting conditions
Sono et al, 2025	• The pilot study assessed patients' understanding of key terms including antibiotics and AMR when leaving 11 community pharmacies<br>• Additionally, patients were asked if any antibiotics they left with were formally prescribed or merely dispensed	• Eleven out of 15 interviewed patients received antibiotics, of these, eight did not have a prescription.<br>• Among patients dispensed an antibiotic with a prescription – the majority (66.7%) were for URTIs with 33.3% for STIs<br>• STIs (50%) were the most prevalent indication when antibiotics were dispensed without a prescription with limited dispensing of antibiotics without a prescription for URTIs (12.5%)
Van Hecke et al, 2025	• The objective was to assess the impact of a pharmacist-prescriber partnership to review antibiotic prescribing in public PHCs<br>• 457 patients with acute coughs were enrolled at 5 PHCs.	• 84% of patients enrolled in the review were prescribed an antibiotic for their acute cough<br>• The most prescribed antibiotics for these patients were amoxicillin (63%), co-amoxiclav (13%) and phenoxymethylpenicillin (6%), with a diagnosis of 'community-acquired pneumonia' as the principal indication (35%)<br>• There was also a significant proportion of patients prescribed an antibiotic for 'acute cough' which needs addressing with future AMS activities including community pharmacists to reduce AMR

NB: ABR = Antibiotic Resistance; AMR = Antimicrobial Resistance; AMS = Antimicrobial Stewardship; ARI = Acute Respiratory Infection; AWaRe = Access, Watch and Reserve (Sharland et al., 2019); EML = Essential Medicines List; GPs = General Practitioners; PHCs = Primary Healthcare Clinics;

PPS = Point Prevalence Survey; RTIs = Respiratory Tract Infections; SPs = Simulated Patients; STGs = Standard Treatment guidelines; STIs = Sexually Transmitted Infections; URTIs = Upper Respiratory Tract Infections; UTIs = Urinary Tract Infection

Supplementary Table S2: Examples of indicators currently used to assess prescribing quality in PHC facilities in South Africa

**Prescribing (quality) indicator/quantity metric**	**References**
% of prescriptions adhering to current guidelines	Matsitse et al., 2017; Gasson et al., 2018; Govender et al., 2021
% of antibiotics prescribed/procured broken down by AWaRe categories	Sharma et al., 2020; Alabi and Essack, 2022; Skosana et al., 2022; Van Hecke et al., 2025
% of patients prescribed an appropriate antibiotic dose and duration for their diagnosed infectious disease	De Vries et al., 2022
% of patients prescribed an antibiotic (empirically) for an ARI/URTI	Ncube et al., 2017; Mathibe and Zwane, 2020; Guma et al., 2022
% of adherence to antibiotic prescribing process measures (e.g., allergies documented, diagnoses provided, appropriate prescribing according to current guidelines, appropriate doses of antibiotics prescribed, their frequency and duration, as well as a valid prescription	De Vries et al., 2022
% of monthly antibiotics used (defined daily doses per 100 prescriptions dispensed)	De Vries et al., 2022

NB: AWaRe = Access, Watch, Reserve (Sharland et al, 2019); ARI = acute respiratory illness; URTI = upper respiratory tract infection.

Supplementary Table S3 – Overview of the global and South African methods for developing quality indicators

	**Global indicators**	**South African indicators**
Overview of the RAM	The Research and Development (RAND)/University of California Los Angeles (UCLA) appropriateness method (RAM) originally developed in the 1950s, is a commonly used method to identify consensus, combining a synthesis of the evidence base with expert opinions (Brown, 1968; Fitch et al., 2001; Campbell et al., 2002). The RAM technique is ideal for developing QIs because it uses a series of intensive ratings sheets combined with controlled aggregate feedback and interactive discussion of the proposed QIs (Fitch et al., 2001).	
**Identification of potential indicators**	Indicators were identified though a literature search and reviewed to focus on the clinical infections and guidance in the WHO AWaRe Antibiotic Book (Sharland et al., 2022; Zanichelli et al., 2023).	Fifty-six primary care indicators and six general indicators were adapted from the global RAM. One indicator was adapted from the global Delphi Technique. Fifteen additional indicators were developed specifically for the South African context based on results from the Antibiotic Prescribing in Primary Healthcare Point Prevalence Survey (APC-PPS) conducted at eight PHC clinics in the North-West and Gauteng provinces in South Africa between November 2023 and October 2024 (Chigome et al., 2026; Cook et al., 2026). The key findings from the APC-PPS showed that acute cough and sexually transmitted infections (STIs) were the most common infection presentations, warranting the need for the additional indicators to assess the appropriateness of antibiotic prescribing for these infection presentations, aligned with the AWaRe Antibiotic Book (Sharland et al., 2022; Zanichelli et al., 2023; Chigome et al., 2025).
**Consensus methodology**	Two distinct methodologies used: the first was a two-round Delphi Technique, engaging a panel of over 100 international experts to rate the indicators for appropriateness and feasibility of the indicators within national and local health system contexts (Khodyakov et al., 2023; Heath et al., 2025). The second was a separate RAM comprising a panel of 12 global experts in AMR/AMS from all the WHO regions, focusing specifically on evaluating the appropriateness of the indicators in a global context (Heath et al., 2025 and 2026).	The South African study used the RAM to develop quality indicators specific to public PHC settings (Fitch et al., 2001; Chigome et al., 2026). The first round of the RAM had 12 multidisciplinary national panel members, and the second round had 10 panel members (Chigome et al., 2026).
**No. of indicators rated**	The Delphi Technique included 102 indicators (Primary Care: 46; Hospital: 39; General:17). The RAM resulted in 136 indicators (Primary Care: 56;	78 indicators were rated in the first round of the RAM, and 89 indicators were rated in the second round of the RAM. Both rounds included the 62 indicators adapted from the global RAM (Chigome et al., 2026).

	Hospital: 60, General: 20) (Heath et al., 2025 and 2026).	
**Data analysis**	The median ratings and level of consensus were calculated in an identical way for each indicator for clarity and appropriateness in the first round and for appropriateness and feasibility in the second round, for both sets of indicators (Chigome et al., 2026; Heath et al., 2026).	

Supplementary Table S4 – Definitions and levels of appropriateness, feasibility and consensus [Chigome et al., 2026; Heath et al., 2026]

	**Definitions**
**Key terms**	
**Appropriateness**	The extent to which an indicator is beneficial, effective, and evidence-based (or clinically indicated) when applied to the 'average' patient in primary care
**Feasibility**	The practicality of implementing the indicator in routine clinical practice (i.e., is it feasible from a human, data, workforce and financial perspective in the South African context?)
**Clarity**	The degree to which the indicator is clearly and precisely defined, unambiguous, and easily understood by clinicians and stakeholders
**Rating scores for appropriateness and feasibility (9-point Likert scale)**	
**1**	Completely inappropriate/infeasible (no exceptions ever)
**2**	Very inappropriate/infeasible: rare exceptions
**3**	Inappropriate/infeasible: some exceptions
**4**	Equivocal but inappropriate/infeasible for many
**5**	Equivocal
**6**	Equivocal but appropriate/feasible for some
**7**	Appropriate/feasible: some exceptions
**8**	Very appropriate/feasible: rare exceptions
**9**	Completely appropriate/feasible (no exceptions ever)
**Levels of appropriateness and feasibility**	
**1 - 3**	Inappropriate/infeasible
**4 - 6**	Equivocal
**7 - 9**	Appropriate/feasible
**Levels of consensus**	
**Agreement (A)**	≥80% rated within +/- 1 of the median
**Disagreement (D)**	≥ 33% rated in both 1-3 and 7-9 score ranges
**Equivocal (E)**	Neither disagreement nor agreement (no consensus)

References

Alabi, M.E, Essack, S.Y. (2022). Antibiotic prescribing amongst South African general practitioners in private practice: an analysis of a health insurance database. *JAC Antimicrob Resist* 4(5):dlac101. doi: 10.1093/jacamr/dlac101

Brown, B. Delphi Process: A Methodology Used for the Elicitation of Opinions of Experts; RAND Corporation: Santa Monica, CA, USA, 1968; Available online: https://www.rand.org/pubs/papers/P3925.html (accessed on 21 March 2026)

Campbell, S.M., Braspenning, J., Hutchinson, A., Marshall, M. Research methods used in developing and applying quality indicators in primary care. Qual Saf Health Care. 2002;11(4):358-64. doi: 10.1136/qhc.11.4.358

Chigome, A., Vhambe, S., Kganyago, K., Meyer, J., Campbell, S., Godman, B., et al. (2025). Point prevalence surveys of acute infection presentation and antibiotic prescribing in selected primary healthcare facilities in North-West and Gauteng provinces of South Africa. *Int. J. Infect. Dis.* 152, 107689. doi:10.1016/j.ijid.2024.107689

Chigome, A.K., Meyer, J.C., Brink, A., Essack, S., Bronkhorst, E., Dawood, H., et al. (2026). Development of AWaRe-Based Quality Indicators to Assess the Appropriateness of Antibiotic Prescribing in Primary Healthcare in South Africa. *Antibiotics* 15(2):196. doi: 10.3390/antibiotics15020196

Cook, A., Goelen, J., Moore, C.E., Martin, J., Pouwels, K.B., Sharland, M. A pilot protocol for surveillance of infection and antibiotic prescribing in primary healthcare across the globe: Antibiotic Prescribing in Primary Healthcare Point Prevalence Survey (APC-PPS). Wellcome Open Res. 2026 Mar 31;10:26. doi: 10.12688/wellcomeopenres.23420.2.

De Vries, E., Johnson, Y., Willems, B., Bedeker, W., Ras, T., Coetzee, R., et al. (2022). Improving primary care antimicrobial stewardship by implementing a peer audit and feedback intervention in cape town community healthcare centres. *S Afr. Med. J.* 112 (10), 812–818. doi:10.7196/SAMJ.2022.v112i10.16397

Farley, E., Stewart, A., Davies, M.A., Govind., M, Van den Bergh, D., Boyles TH (2018). Antibiotic use and resistance: Knowledge, attitudes and perceptions among primary care prescribers in South Africa. *S Afr Med J*. 108(9):763-71

Fitch, K., Bernstein María, S.J., Aguilar, D., Burnand, B., Lacalle, J.R., Lázaro, P., et al. The RAND/UCLA Appropriateness Method User's Manual; The RAND Corporation: Santa Monica, CA, USA, 2001; Available online: https://www.rand.org/pubs/monograph_reports/MR1269.html (accessed on 21 March 2026)

Gasson, J., Blockman, M., and Willems, B. (2018). Antibiotic prescribing practice and adherence to guidelines in primary care in the cape town metro district, South Africa. *S Afr. Med. J.* 108 (4), 304–310. doi:10.7196/SAMJ.2017.v108i4.12564

Govender, T., Suleman, F., Perumal-Pillay, V.A. (2021). Evaluating the implementation of the standard treatment guidelines (STGs) and essential medicines list (EML) at a public South African tertiary institution and its associated primary health care (PHC) facilities. *J Pharm Policy Prac*t 14(1):105. doi: 10.1186/s40545-021-00390-z

Guma, S. P., Godman, B., Campbell, S. M., and Mahomed, O. (2022). Determinants of the empiric use of antibiotics by general practitioners in South Africa: observational, analytic, cross-sectional study. *Antibiotics* 11 (10), 1423. doi:10.3390/ antibiotics11101423

Heath, A., Goelen, J., Chuki, P., Cook, A., Djukic, F., Thuy Do. N.T., et al. (2025). Development of AWaRe antibiotic quality indicators for optimal use. *JAC Antimicrob Resist*. 2025 Dec 4;7(Suppl 4):dlaf230.006. doi: 10.1093/jacamr/dlaf230.006

Heath, A., Goelen, J., Chuki, P., Cook, A., Djukic, F., Do, N.T.T, et al. (2026) Development of AWaRe antibiotic quality indicators for optimal use. medRxiv preprint - https://www.medrxiv.org/content/10.1101/2025.10.24.25338539v2 (Accessed 15 May 2026)

Keuler, N., Johnson, Y., Coetzee, R. (2022). Treating urinary tract infections in public sector primary healthcare facilities in Cape Town, South Africa: A pharmaceutical perspective. *S Afr Med J*. 112(7):487-493.

Khodyakov, D., Grant, S., Kroger, J., Bauman M. (2023). RAND Methodological Guidance for Conducting and Critically Appraising Delphi Panels. Available at URL: https://www.rand.org/content/dam/rand/pubs/tools/TLA3000/TLA3082-1/RAND_TLA3082-1.pdf (Accessed 10 January 2026)

Lagarde, M., and Blaauwm, D. (2023). Levels and determinants of overprescribing of antibiotics in the public and private primary care sectors in South Africa. *BMJ Glob. Health* 8 (7), e012374. doi:10.1136/bmjgh-2023-012374

Maluleke, T.M., Maluleke, M.T., Ramdas, N., Jelić, A.G., Kurdi, A., Chigome, A., et al. (2025a). Prevalence and Associated Factors for Purchasing Antibiotics Without a Prescription Among Patients in Rural South Africa: Implications for Addressing Antimicrobial Resistance. *Antibiotics* 14(12):1273. doi: 10.3390/antibiotics14121273.

Mathibe, L.J., Zwane, N.P. (2020). Unnecessary antimicrobial prescribing for upper respiratory tract infections in children in Pietermaritzburg, South Africa. *Afr Health Sci* 20(3):1133-1142. doi: 10.4314/ahs.v20i3.15

Matsitse, T.B., Helberg, E., Meyer, J.C., Godman, B., Massele, A., Schellack, N. (2017). Compliance with the primary health care treatment guidelines and the essential medicines list in the management of sexually transmitted infections in correctional centres in South Africa: findings and implications. *Expert Rev Anti Infect Ther* 15(10):963-972. doi: 10.1080/14787210.2017.1382354.

Ncube, N.B.Q., Solanki, G.C., Kredo, T., Lalloo, R. (2017). Antibiotic prescription patterns of South African general medical practitioners for treatment of acute bronchitis. *S Afr Med* 107(2):119-122. doi: 10.7196/SAMJ.2017.v107i2.11276

Sharland, M., Gandra, S., Huttner, B., Moja, L., Pulcini, C., Zeng, M., et al. (2019). Encouraging AWaRe-ness and discouraging inappropriate antibiotic Use—The new 2019 essential medicines list becomes a global antibiotic stewardship tool. *Lancet Infect*. Dis. 19 (12), 1278–1280. doi:10.1016/S1473-3099(19)30532-8

Sharland, M., Zanichelli, V., Ombajo, L.A., Bazira, J., Cappello, B., Chitatanga, R., et al. (2022). The WHO essential medicines list AWaRe book: from a list to a quality improvement system. *Clin Microbiol Infect* 28(12):1533-1535. doi: 10.1016/j.cmi.2022.08.009

Sharma, S., Tandlich, R., Docrat, M., Srinivas, S. (2020). Antibiotic procurement and ABC analysis for a comprehensive primary health care clinic in the Eastern Cape province, South Africa. *S Afr J Infect Dis* 35(1):134. doi: 10.4102/sajid.v35i1.134

Skosana, P.P., Schellack, N., Godman, B., Kurdi, A., Bennie, M., Kruger, D., et al. (2022). A national, multicentre web-based point prevalence survey of antimicrobial use in community healthcare centres across South Africa and the implications. *Hosp Pract* 50(4):306-317. doi: 10.1080/21548331.2022.2114251

Sono, T.M., Mboweni, V., Jelić, A.G., Campbell, S.M., Marković-Peković, V., Ramdas, N., et al. (2025). Pilot Study to Evaluate Patients' Understanding of Key Terms and Aspects of Antimicrobial Use in a Rural Province in South Africa Findings and Implications. *Adv Hum Biol* 15(1):108-12.

Truter, I., and Knoesen, B. C. (2018). Perceptions towards the prescribing of antibiotics by pharmacists and the use of antibiotics in primary care in South Africa. *J. Infect. Dev. Ctries.* 12 (2), 115–119. doi:10.3855/jidc.9630. doi 10.4103/aihb.aihb_119_24

van Hecke, O., Butler, C., Mendelson, M., Tonkin-Crine, S. (2019). Introducing new point-of-care tests for common infections in publicly funded clinics in South Africa: a qualitative study with primary care clinicians. *BMJ Open*. 9(11):e029260. doi: 10.1136/bmjopen-2019-029260.

van Hecke, P. O., Adegoke, D. Y., von Pressentin, P. K., Namane, P. M., Mendelson, P. M., Butler, P. C., et al. (2025). Impact of pharmacist-prescriber partnerships to track antibiotic prescribing in publicly funded primary care in the Cape Town Metropole, South Africa: an implementation study. *Int. J. Infect. Dis.* 152, 107626. doi:10.1016/j.ijid.2024.107626

Wieters, I., Johnstone, S., Makiala-Mandanda, S., Poda, A., Akoua-Koffi, C., Abu Sin, M., et al. (2024). Reported antibiotic use among patients in the multicenter ANDEMIA infectious diseases surveillance study in sub-saharan Africa. *Antimicrob Resist Infect Control*. 13(1):9. doi: 10.1186/s13756-024-01365-w

Zanichelli V, Sharland M, Cappello B, Moja L, Getahun H, Pessoa-Silva C, et al. (2023). The WHO AWaRe (Access, Watch, Reserve) antibiotic book and prevention of antimicrobial resistance. *Bull World Health Organ* 101:290–296 | doi: http://dx.doi.org/10.2471/BLT.22.288614
